# Linear indices of ventricular volume on brain computed tomography as markers of effectiveness of epidural blood patch for spontaneous intracranial hypotension: A case report

**DOI:** 10.1097/MD.0000000000029279

**Published:** 2022-08-12

**Authors:** So Young Lee, Bum Young Park, Taeha Ryu, Ji Hyeon Lee, Dong Hyuck Kim, Woon Seok Roh

**Affiliations:** Department of Anesthesiology and Pain Medicine, Daegu Catholic University Medical Center, School of Medicine, Daegu Catholic University, Nam-gu, Daegu, Republic of Korea.

**Keywords:** case report, computed tomography, epidural blood patch, spontaneous intracranial hypotension, ventricular volume indices

## Abstract

**Rationale:**

Epidural blood patch (EBP) is an effective treatment for spontaneous intracranial hypotension (SIH). However, its effectiveness can only be judged through subjective symptom improvement; no objective markers have been reported. Linear indices of ventricular volume on brain computed tomography (CT) may aid the objective evaluation of the effectiveness of EBP in patients with SIH.

**Patient concerns:**

A 45-year-old man was hospitalized due to a 3-week history of orthostatic headache, dizziness, and neck pain. He had visited a local emergency department at symptom onset. His neurological examination results were normal and vital signs were stable.

**Diagnoses:**

Brain magnetic resonance imaging (MRI) revealed pachymeningeal enhancement in both convexities with a small subdural hematoma (SDH). Based on the clinical features and MRI findings, he was diagnosed with SIH complicated by SDH.

**Interventions:**

Non-targeted EBP was performed, first at the lumbar level and subsequently at the thoracic level. Linear indices of ventricular volume, including the Evans’ index, frontal–occipital horn ratio, and bicaudate index, were measured through brain CT performed before and after EBP.

**Outcomes:**

After lumbar EBP, there was no symptom relief or increase in linear indices of ventricular volume on brain CT. In contrast, the patient’s symptoms completely resolved and the linear indices of ventricular volume increased after thoracic EBP.

**Lessons:**

The effectiveness of EBP, which is currently evaluated solely based on changes in symptom severity, can be assessed using linear indices of ventricular volume.

## 1. Introduction

Spontaneous intracranial hypotension (SIH) is characterized by headache that worsens in an upright position due to cerebrospinal fluid (CSF) leakage in the absence of a trauma history and an iatrogenic cause.^[1]^ Epidural blood patch (EBP) is effective for treating SIH that does not resolve with conservative treatment, which includes bed rest, hydration, and analgesic agent administration.^[2]^ When EBP is performed, the dural defect is directly sealed, and an immediate effect is observed through compression of the dural sac.^[1,3]^ The effectiveness of EBP is generally assessed based on symptom improvement; no objective method for its evaluation has been reported. Here, we report a case of SIH in which the increase in ventricular volume after EBP was evaluated on brain computed tomography (CT) using linear indices of ventricular volume, including the Evans’ index, frontal–occipital horn ratio (FOHR), and bicaudate index.

## 2. Ethics statement

Written informed consent was obtained from the patient for the publication of this report.

## 3. Case report

**Figure 1.**
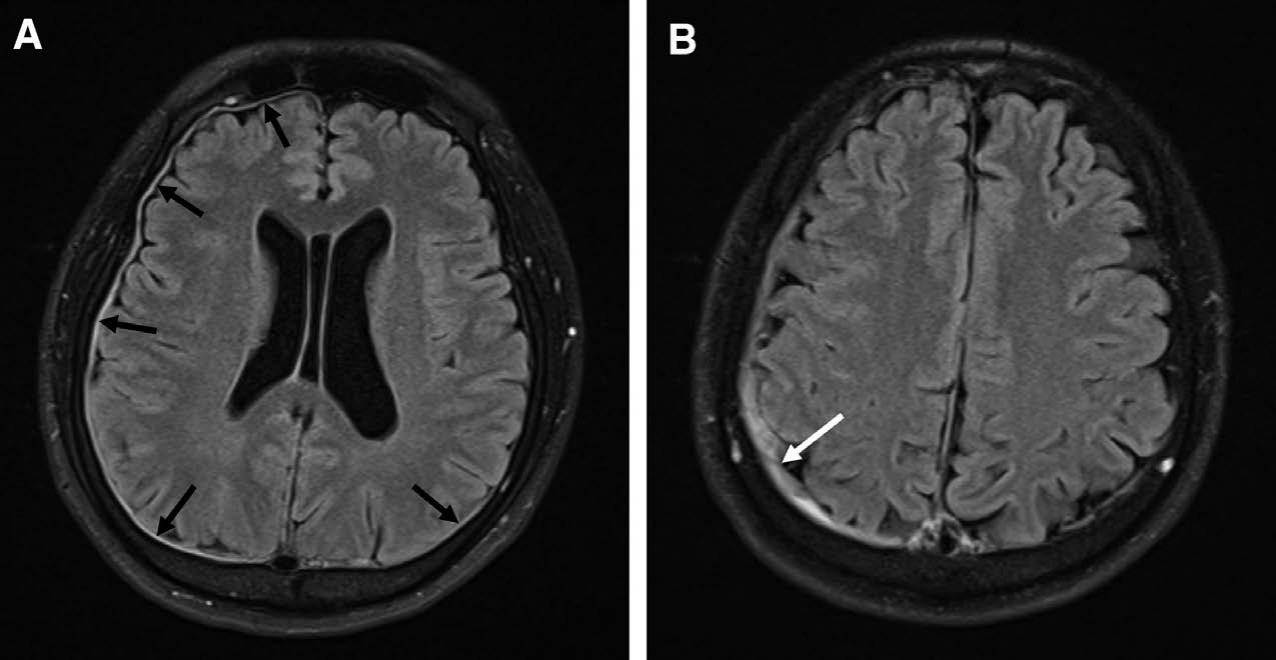
MRI images showing pachymeningeal enhancement and SDH. Contrast-enhanced T1-weighted MRI images shows diffuse pachymeningeal enhancement (A, black arrows), which is a radiological finding characteristic of SIH, and a small SDH (B, white arrow). MRI = magnetic resonance imaging, SIH = spontaneous intracranial hypotension, SDH = subdural hematoma.

**Figure 2.**
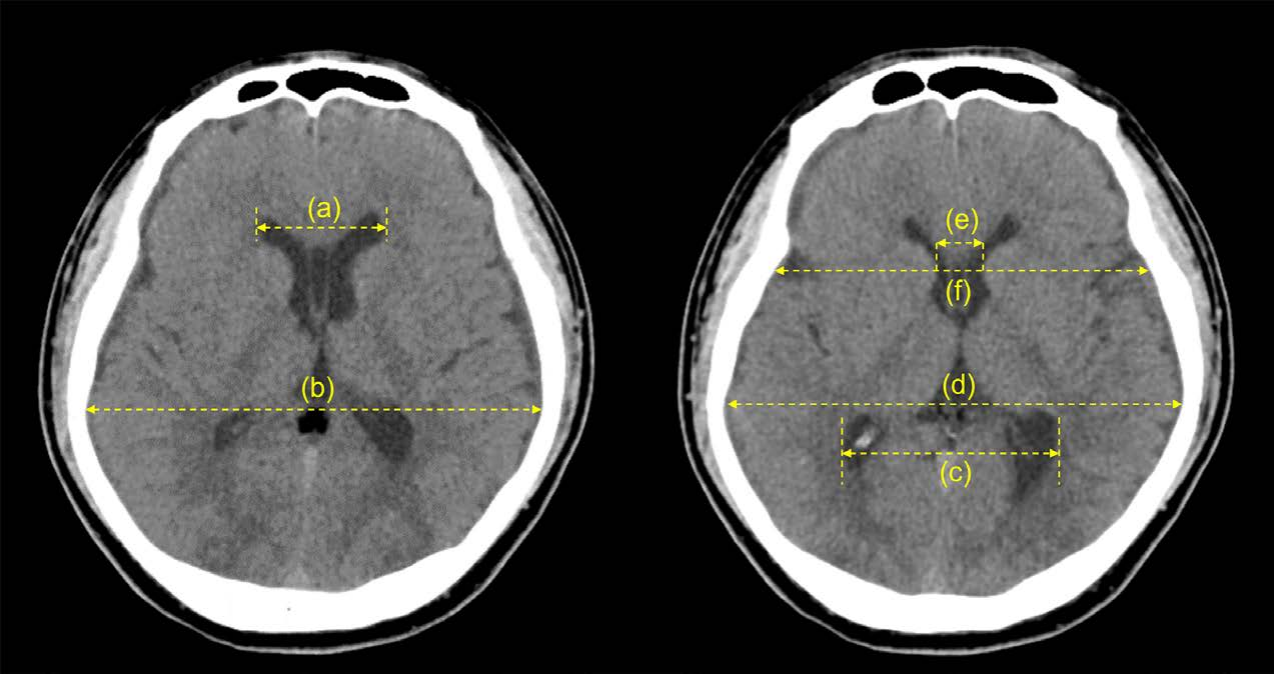
Schematic representation of the measurement of the linear indices of ventricular volume. (a) Maximum frontal horn width. (b) Maximum internal diameter of the skull in the slice in which (a) is measured. (c) Maximum occipital horn width. (d) Maximum internal diameter of the skull. (e) Minimum intercaudate distance. (f) Internal diameter of the skull at the place where (e) is measured. Evans’ index is the maximum width of the frontal horn divided by the maximum internal diameter of the skull in the same slice.^[4]^ Frontal-occipital horn index is the average of the maximum width of the frontal and lateral horns divided by the maximum internal diameter of the skull.^[5]^ Bicaudate index is the minimum intercaudate distance divided by the internal diameter of the skull at the place where the intercaudate distance is measured.^[6]^

A 45-year-old otherwise healthy man with no recent history of trauma or lumbar puncture was hospitalized due to orthostatic headache. Three weeks before admission, he had visited the emergency department of another institution due to postural headache, dizziness, and neck pain. The headache was predominantly at the occiput, and the visual analog scale (VAS) for headache intensity was 6. The neurological examination results were normal and vital signs were stable. Brain magnetic resonance imaging (MRI) revealed pachymeningeal enhancement in both convexities and a small subdural hematoma (SDH; Fig. 1). The patient was diagnosed with SIH complicated by SDH. Because the SDH was small, the neurosurgeon opted to perform nonoperative management and surveillance with brain CT. For treating the SIH, the patient was advised bed rest and prescribed analgesics such as acetaminophen and non-steroidal anti-inflammatory drugs. However, the symptoms were not relieved. When the patient was transferred to our hospital, we found that no radiological examination had been performed to locate the site of CSF leakage. Therefore, we decided to perform non-targeted EBP. We performed EBP at the L4–L5 intervertebral level with 15 mL of autologous blood, following which the patient was prescribed bed rest for 24 hours. Nevertheless, the VAS for headache intensity remained 6. We measured the linear indices of ventricular volume using previously described techniques^[4–6]^ (Fig. 2). There were no differences between the pre- and post-EBP values of the Evans’ index, FOHR, and bicaudate index measured on axial brain CT (Table 1). Because there was no improvement in symptoms and linear indices of ventricular volume, instead of repeating the lumbar EBP, we performed EBP at the T1–T2 intervertebral level with 15 mL of autologous blood. The VAS for headache intensity reduced to 2. Brain CT was performed 1 day pre- and post-EBP, and a comparison of the results showed that the Evans’ index, FOHR, and bicaudate index had increased by 7.41%, 7.89%, and 18.18%, respectively, in addition to improvement in the VAS score (Table 1). Five days after thoracic EBP, the patient’s symptoms had completely resolved and he was discharged home. One week after discharge, he had no symptoms and brain CT revealed no decrease in the linear indices of ventricular volume.

**Table 1:** Pre- and post-intervention values of the linear indices of ventricular volume.

	Pre–lumbar EBP	Post–lumbar EBP	Pre–thoracic EBP	Post–thoracic EBP	Final follow-up CT
(a) (mm)	38.46	38.30	38.10	40.50	41.93
(b) (mm)	140.89	139.96	139.37	139.83	139.60
(c) (mm)	69.29	68.75	68.47	73.29	73.20
(d) (mm)	140.89	139.96	139.60	140.08	139.99
(e) (mm)	13.44	13.27	13.02	14.95	14.71
(f) (mm)	115.12	115.10	115.81	115.25	115.02
Evans’ index (a)/(b)	0.27	0.27	0.27	0.29	0.30
FOHR [(a)+ (c)]/2(d)	0.38	0.38	0.38	0.41	0.41
Bicaudate index (e)/(f)	0.12	0.12	0.11	0.13	0.13

## 4. Discussion

EBP is the mainstay of SIH treatment. It can be categorized as targeted or non-targeted EBP. Targeted EBP involves extensive diagnostic imaging to detect the site of leakage and to determine where blood should be injected. It is considered more effective than non-targeted EBP.^[7]^ Therefore, some physicians prefer to locate the site of leakage before performing EBP. However, non-targeted EBP, also known as speculative or empiric EBP, is commonly performed because identifying the exact site of CSF leakage in patients with SIH is challenging. Spinal imaging techniques such as CT or magnetic resonance myelography with intrathecal contrast agent and radionuclide cisternography are used to locate the leakage site.^[8,9]^ However, the performance of these modalities involves dural puncture, which is invasive, and there are concerns regarding neurotoxicity induced by the intrathecal contrast agent.^[2,10]^ A recent systematic review demonstrated similar success rates for targeted and non-targeted EBP.^[2]^ This is why not all physicians advocate initial targeted EBP. Conventionally, non-targeted EBP is first performed at the lumbar level despite the thoracic spine being the most common site of CSF leakage.^[11,12]^ When lumbar EBP is performed, the injected blood can spread from the lumbar spine to the cervical spine.^[13]^ In addition, complications such as cord injury are less likely to occur at the lumbar level than at other levels.^[12,14]^ If symptoms are not relieved, the lumbar EBP can be repeated or EBP can be performed at higher vertebral body levels, such as thoracic levels. Other techniques of non-targeted EBP, such as bilevel and multilevel EBP, have recently been reported to be more effective than the conventional non-targeted EBP technique.^[15,16]^ In these techniques, clinicians simultaneously inject blood at multiple levels, which leads to the spread of blood throughout the epidural space. If the patient shows no response to non-targeted EBP, the abovementioned imaging modalities should be used to identify the target site,^[2]^ and other etiologies of headache should be simultaneously explored. Orthostatic headache can be caused by postural orthostatic tachycardia syndrome, cervicogenic headache, and craniocervical instability.^[1]^

In this case, we focused on objective findings that may indicate the effectiveness of EBP in patients with SIH. Brain CT is not routinely performed before and after EBP. However, to document the stability of the SDH, our patient underwent brain CT whenever there was any change in treatment. Therefore, imaging data before and after the procedures were available. We hypothesized that if the blood clot sealed the CSF leakage site after EBP, the ventricular volume on brain CT would increase within 1 to 2 days. Several indices have been recommended for detecting changes in the ventricular volume.^[5,17,18]^ The Evans’ index is the most commonly used index for evaluating ventricular enlargement, especially for diagnosing idiopathic normal-pressure hydrocephalus.^[19]^ The bicaudate index and FOHR are linear indices that are measured during the follow-up of patients with ventriculomegaly.^[5,18]^

This case shows that the linear indices of ventricular volume did not increase when there was no symptomatic improvement after the first EBP at the lumbar level. In contrast, thoracic EBP, which was the second intervention, produced both symptom relief and an increase in the linear indices of ventricular volume. These findings suggest that linear indices of ventricular volume on brain axial CT can be used as an objective indicator of the effectiveness of EBP in patients with SIH. In addition to symptom improvement, these indices can help clinicians decide whether to re-attempt EBP at the lumbar level or perform it at the thoracic level, which is particularly useful when performing non-targeted EBP. However, the use of linear indices of ventricular volume has some limitations. Even though linear indices have been reported to be correlated with ventricular volume, they are indirect markers of ventricular volume.^[4,6]^ The lateral ventricles are three dimensional, but each linear index is measured on a two-dimensional axial brain CT slice.^[19]^ This can decrease their reliability. Novel MRI-based methods for measuring actual ventricular volume have been developed. Their accuracy is assured. However, direct volumetric assessment requires the use of special software with an automated measuring system; manual measurement can be performed, but it consumes a significant amount of time.^[17]^ In contrast, the method of using linear indices to assess changes in ventricular volume is simple, quick, reproducible, and easy to perform in a clinical setting. This makes it a good alternative to direct volumetric assessment.

We believe that this case report is interesting because we assessed the outcome of EBP based on the change in ventricular volume. Comparing linear indices of ventricular volume on brain CT before and after EBP can help clinicians detect volumetric changes in the CSF system and ascertain the effectiveness of EBP. Further research and accumulation of cases are needed to confirm the usefulness of these indices. Moreover, the sensitivity of each index should be investigated.

## Author contributions

Conceptualization: Woon Seok Roh

Data curation: Bum Young Park, Dong Hyuck Kim, Ji Hyeon Lee, So Young Lee, Woon Seok Roh

Formal analysis: Bum Young Park, So Young Lee, Woon Seok Roh

Visualization: So Young Lee

Writing – original draft: So Young Lee, Woon Seok Roh

Writing – review & editing: So Young Lee, Taeha Ryu, Woon Seok Roh
